# Foam‐Mat Drying of Camel Milk: Evaluation of Physicochemical Quality Attributes and Powder Properties

**DOI:** 10.1002/fsn3.72084

**Published:** 2026-07-07

**Authors:** Mohamad Ali Podine, Mohammd Nejatian, Morteza Fathi, Maryam Taghdir, Najmeh Youseftabar Miri

**Affiliations:** ^1^ Student Research Committee Baqiyatallah University of Medical Sciences Tehran Iran; ^2^ Department of Nutrition Science and Food Hygiene, Faculty of Health Baqiyatallah University of Medical Sciences Tehran Iran; ^3^ Health Research Center, Life Style Institute Baqiyatallah University of Medical Sciences Tehran Iran

**Keywords:** drying kinetics, organoleptic characteristics, physical properties, response surface methodology

## Abstract

Camel milk is a nutritious but highly perishable dairy product, necessitating effective preservation methods to extend its shelf life and enable wider distribution while preserving its nutritional value. This study aimed to optimize the production of camel milk powder using the energy‐efficient foam‐mat drying technique. In this process, camel milk was mixed with whey protein concentrate (WPC) and gum tragacanth (GT) dispersion to form a stable foam. The foam formulation was optimized via Response Surface Methodology (RSM), with WPC (5%–15% w/w), GT (0.05%–0.2% w/w), and whipping time (1–4 min) as independent variables. Quadratic models for foam density and stability were significant, identifying WPC as the most influential factor. The optimized foam was dried at 50°C, 60°C, and 70°C. Drying kinetics were best described by the Page model (*R* ^2^ > 0.99). The powder dried at 70°C exhibited superior quality: the lowest water activity (0.136), highest solubility (0.88), lowest bulk density (0.45 g/cm^3^), and highest lightness (L* = 89.39). Hygroscopicity increased slightly with temperature but remained within acceptable limits for dairy powders. Sensory evaluation showed no significant differences among samples, though the 70°C powder received the highest scores for taste (mean = 4.60) and overall acceptability (mean = 4.12). In conclusion, foam‐mat drying at 70°C effectively produces a high‐quality, shelf‐stable, and sensorially acceptable camel milk powder, offering a promising alternative to conventional drying methods.

## Graphical abstract

Camel milk was mixed with whey protein concentrate and gum tragacanth to form a stable foam. The foam was dried at 50°C, 60°C, and 70°C using the foam‐mat drying technique. The powder produced at 70°C exhibited the lowest water activity (0.136), highest solubility (0.88), and desirable physicochemical properties, making it a promising shelf‐stable product.

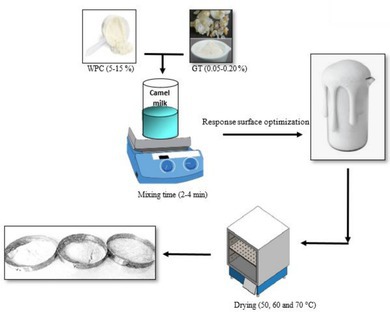

## 1 Introduction

Camel milk has gained increasing attention in recent years due to its unique nutritional and therapeutic properties compared with bovine milk. It contains healthier lipid profile (decreased cholesterol and saturated fatty acids in conjunction with increased essential fatty acid levels) (Bakry et al. 2021) and higher levels of vitamin C, some mineral content (Zn, Cu, Fe, and Mn) and bioactive proteins (such as lactoferrin, lysozyme, and immunoglobulins), which have been associated with antioxidant, antimicrobial, and antidiabetic effects (Alhassani 2024; Swelum et al. 2021). For example, during 3 weeks of trial (250 mL daily consumption), there was a 210% decrease in the mean blood glucose level of the treated rats getting raw camel milk (Agrawal et al. 2004). In mice with dextran sodium sulfate‐induced colitis, it has also demonstrated promising effects in improving gut health by modifying the intestinal microbiota and lowering inflammatory indicators (He et al. 2022). Beyond its nutritional value, camel milk plays a significant role in achieving several United Nations Sustainable Development Goals (SDGs), including poverty alleviation, food security, gender equality, and climate resilience (Chikha and Faye 2025).

Owing to these functional attributes, camel milk has been recognized as a promising functional food, particularly for populations in arid and semi‐arid regions where camels are a primary source of nutrition (Swelum et al. 2021). However, its high perishability and susceptibility to microbial spoilage limit its shelf life and restrict its broader commercialization. Therefore, the development of stable powdered forms of camel milk has become an essential strategy for extending its shelf life, improving transportation and storage, and expanding its use in functional food formulations (Ho et al. 2019).

Various drying techniques, including spray drying and freeze drying, have been employed for producing camel milk powder (Gebrehiwot and Banat 2025; Rakhmatulina et al. 2024). Although freeze drying preserves bioactive compounds effectively, it is energy‐intensive and costly (Ibrahim and Khalifa 2015). Spray drying, on the other hand, offers scalability but may cause heat‐induced denaturation of sensitive proteins and loss of volatile nutrients (Kaskous 2016). Foam‐mat drying has recently emerged as a promising alternative drying method for liquid foods, in which a stable foam is formed by whipping the product with suitable foaming agents, followed by drying at relatively low temperatures. This technique provides advantages such as faster drying rates, lower energy consumption, and better retention of heat‐sensitive nutrients and functional compounds (Sangamithra et al. 2015).

Despite the increasing interest in camel milk, limited studies have explored the use of foam‐mat drying for its dehydration. Most available literature has focused on conventional drying techniques, leaving a knowledge gap regarding how foam‐mat drying affects the physicochemical, functional, and nutritional properties of camel milk powder. Evaluating these properties is critical to determine the feasibility of this technique for producing high‐quality powders that retain the nutritional value of fresh camel milk. The present work is one of the first attempts to investigate the physicochemical characteristics of camel milk powder produced by foam‐mat drying. By assessing key parameters such as bulk density, solubility, water activity, color, and reconstitution properties, this study aims to provide novel insights into optimizing the processing and application of camel milk powder in the functional food industry.

## 2 Materials and Methods

### 2.1 Materials

Raw camel milk was freshly ordered and purchased from a camel milk production and distribution company in Gorgan (Golestan province of Iran). The milk was transported to the laboratory within 2 h after milking in insulated containers at 4°C and was processed within 24 h of collection. According to the supplier's specifications, the total microbial count of the milk was 3 × 10^5^ CFU/mL. Gum tragacanth (GT) (*Astragalus gossypinus*) was bought from a local herbal store. Cow whey protein concentrate (WPC) was supplied by DMV International (Veghel, Netherlands).

### 2.2 Preparation of GT Dispersion

The raw GT was pulverized in a high‐speed mechanical blender and sieved to obtain uniform samples with a mesh size between 200 and 500 μm. The required amount of GT powder was weighed, and it was then gradually (over the course of an hour) added to a beaker containing deionized water on a magnetic stirrer to obtain uniform dispersion. Afterwards, to ensure complete hydration, the dispersion was kept for 24 h at 5°C to allow full swelling of GT polysaccharides (Hatami et al. 2012, 2014), without periodic stirring.

### 2.3 Experimental Design

Response surface methodology (RSM) was used to evaluate the effects of independent variables (foaming agent [WPC] concentration 5%–15% w/w [*X*_1_], stabilizing agent [GT] concentration 0.05%–0.2% w/w [*X*_2_], and the whipping time [WT] 1–4 min [*X*_3_]) as well as their interactions on responses (foam density [*Y*_1_] and foam stability [*Y*_2_]). The selected ranges were determined based on preliminary trials (data not shown). The coded and uncoded independent variables used in the RSM design are shown in Table 1. As seen in Table 2, the experiments were set up using the central composite design (CCD) with six central points. The responses were expressed as a function of the independent variables using a second‐order polynomial equation:

$$\begin{array}{cc} Y_{i}=\; & a_{0}+a_{1}X_{1}+a_{2}X_{2}+a_{3}X_{3}+a_{11}X_{1}^{2}+a_{22}X_{2}^{2}+a_{33}X_{3}^{2}\\ & +a_{12}X_{1}X_{2}+a_{13}X_{1}X_{3}+a_{23}X_{2}X_{3} \end{array}$$

where *a*_0_ is a constant, *a*_*i*_, *a*_*ii*_, and *a*_*ij*_ are the linear, quadratic, and interactive coefficients, respectively. The coefficients of the response surface equation were determined using Design‐Expert 12 software.

**TABLE 1:** Uncoded and coded independent variables used in RSM design.

Symbol	Independent variable	Coded levels				
		−1.68	−1	0	1	1.68
*X*_1_	WPC (%)	1.59	5	10	15	18.41
*X*_2_	GT (%)	0	0.05	0.13	0.20	0.25
*X*_3_	WT (min)	0.02	1	2.50	4	5.02

**TABLE 2:** Effect of different foam formation conditions (WPC and GT concentrations and whipping time) on foam stability and foam density.

Run	Independent variable^a^			Response^b^	
	*X*_1_	*X*_2_	*X*_3_	*Y*_1_	*Y*_2_
**1**	**15**	**0.2**	**4**	**0.556**	**43.9**
2	5	0/05	4	0.840	2.5
3	10	0.13	2.5	0.747	10
4	15	0.05	4	0.700	15
5	10	0.13	2.5	0.679	24.39
6	10	0.13	5.02	0.846	2.43
7	10	0.13	2.5	0.800	5
8	10	0.13	2.5	0.700	17.5
9	15	0.05	1	0.562	10
10	5	0.05	1	0.875	5
11	10	0.13	0.02	0.663	12.5
12	5	0.2	1	0.540	10
13	10	0	2.5	0.580	10
14	15	0.2	1	0.643	27.5
15	10	0.25	2.5	0.670	35
16	1.59	0.13	2.5	0.960	0
17	5	0.2	4	0.898	5
18	10	0.13	2.5	0.690	20
19	18.41	0.13	2.5	0.780	14.63
20	10	0.13	2.5	0.685	25

*Note:* Each run was performed once as per the CCD design, with six replicates at the central point for pure error estimation. Bold values indicate the optimal foam properties (minimum foam density and maximum foam stability) selected for further drying experiments based on the CCD design.

^a^ *X*_1_: WPC concentration (%); *X*_2_: GT concentration (%); *X*_3_: Whipping time (min).

^b^ *Y*_1_: Foam density (g/cm^3^); *Y*_2_: Foam stability (%).

The Design‐Expert 12 program was used to perform the analysis of variance (ANOVA), calculate regression coefficients, and execute the performance stepwise procedure to simplify the models and create three‐dimensional surface plots. For each response, the significance of the equation parameters was evaluated using the *F*‐value at a probability level of 0.05. Furthermore, the models' adequacy was assessed using model analysis, lack‐of‐fit testing, and coefficient of determination (*R* ^2^) analysis.

### 2.4 Foam Preparation

To produce foam, various amounts of WPC (recommended by CCD design) (80% protein, as per supplier's specification; Pegah Dairy Industry, Iran) were added to camel milk, and after stirring with a magnetic stirrer for about 30 min, the mixture was added to the tragacanth dispersion with a given concentration (prepared as described in Section 2.2). The mixture was allowed to homogenize for an additional 1 h. The final mixture was placed in a water bath with an adjusted temperature of 60°C for 20 min to partially denature whey proteins, thereby enhancing their foaming properties by exposing hydrophobic groups (Kinsella 1981). Then, it cooled with a mixture of water and ice to reach room temperature. Finally, it was stirred with a Saya kitchen blender (model SH‐201, Iran) at the maximum speed for the defined times to produce foam.

### 2.5 Foam Properties

Foam density was determined using the method described by Nejatdarabi et al. (2023). The 50 mL of foam obtained was transferred to the pre‐weighted graduated cylinder with the aid of a spatula and the weight of the foam was then measured and expressed as g/cm^3^ (Nejatdarabi et al. 2023):

$$\text{Foam density}\;\left(g/{\mathrm{cm}}^{3}\right)=\frac{m}{V}$$

where *m* shows 50 mL foam mass and *V* indicates 50 mL foam volume.

The procedure described by Affandi et al. (2017) was used to determine foam stability. The reduction in foam volume after 2 h at room temperature was measured and foam stability was calculated using the following relationship (Affandi et al. 2017):

$$\text{Foam stability}\;\left(\%\right)=\frac{V_{1}}{V_{0}}\times 100$$

where *V*_1_ is the volume of foam after 2 h (cm^3^) and *V*_0_ is the initial volume of foam (cm^3^).

### 2.6 Foam Drying

The prepared foam was poured into aluminum plates (thickness: 3 mm) at approximately 10 mL per plate, and dried with hot air at various air temperatures of 50°C, 60°C, and 70°C (FanAzma, Iran), using a constant air velocity of 1.5 m/s. Samples were weighed every 15 min using an analytical balance (TE214S; Sartorius, Göttingen, Germany). Drying was stopped when the weight change between two consecutive measurements was less than 0.01 g (constant weight). The plates were scraped of dried foam, ground by a kitchen grinder (Saya QMC‐20, Iran), placed in sealed plastic zipper bags, and stored at room temperature (25°C ± 2°C) in a desiccator containing silica gel to avoid moisture absorption prior to quality assessment.

### 2.7 Modeling of the Drying Process

Experimental data for drying curves was gathered as follows: during drying: foam samples were taken from the dryer every 5 min, weighed on a TE214S analytical balance (Sartorius, Göttingen, Germany), and the moisture ratio (MR) was calculated using the following equation:

$$\mathrm{MR}=\left(M_{t}–M_{e}\right)/\left(M_{i}–M_{e}\right)$$

where *M*_*t*_ is moisture content at any time of drying, *M*_*i*_ is initial moisture content and *M*_*e*_ is equilibrium moisture content. *M*_*e*_ was determined by drying the foam samples at 105°C for 24 h until constant weight was achieved. All moisture content values (*M*_*t*_, *Mᵢ*, *M*_*e*_) were calculated on a wet basis.

MR as a function of drying time was fitted using three different thin layer drying models: Page model (Equation 1) (Sarsavadia et al. 1999), Lewis model (Equation 2) (Doymaz 2006), and Henderson‐Pabis model (Equation 3) (Henderson and Pabis 1961). These models were selected because they are the most widely used thin‐layer drying models for food foams and have been successfully applied to dairy and plant‐based foam systems in previous studies (Inyang et al. 2018; Thuy et al. 2025).

(1) $$\begin{array}{c} \mathrm{MR}=e^{-{\mathrm{kt}}^{N}}\\ \ln\left[-\ln\left(\mathrm{MR}\right)\right]\;=\ln\left(k\right)+N\ln\left(t\right) \end{array}$$

where *k* (the drying rate constant [min^−1^]) and *N* (empirical constants of the model) are constants obtained from the *y*‐intercept and the slope of the linear graph ln[−ln(MR)] versus ln(*t*), respectively.

(2) $$\begin{array}{c} \mathrm{MR}=e^{-\mathrm{kt}}\\ \ln\left(\mathrm{MR}\right)\;=-\mathrm{kt}+1 \end{array}$$

(3) $$\begin{array}{c} \mathrm{MR}={\mathrm{ae}}^{-\mathrm{kt}}\\ \ln\left(\mathrm{MR}\right)\;=-\mathrm{kt}+\ln\left(a\right) \end{array}$$

where the drying constants *a* and *k* are obtained from the *y*‐intercept and the slope of the plot of ln(MR) versus *t*, respectively.

The adequacy of the fitted models was evaluated based on two parameters: *R* ^2^ (determination coefficient) and RMSE (root mean squared error). The model with the highest *R* ^2^ value and the lowest RMSE will be selected as the best model to predict the camel milk drying process. Mathematical modeling of the experimental data were performed using the MATLAB software package (R2015a).

### 2.8 Powder Properties

#### 2.8.1 Water Activity

A water activity meter (Rotronic Hygrolab, Switzerland) was used to measure the *a*_w_ of the camel milk powder at 25°C. To achieve this, the water activity was measured after adding a measured amount of powder to the instrument cup.

#### 2.8.2 Hygroscopicity

One gram of each sample was poured onto a glass plate in order to determine its hygroscopicity. The plates were then kept in a desiccator containing 100 mL of saturated NaCl solution at the bottom to maintain a relative humidity of approximately 75% at 25°C. The plates were reweighed after 7 days. Hygroscopicity was calculated using the following formula:

$$\text{Hygroscopicity}\;\left(\%\right)=\left[\left(W_{2}–W_{1}\right)/W_{1}\right]\times 100.$$

where *W*_1_ is the initial weight of the sample (g) and *W*_2_ is the final weight of the sample after 7 days (g).

#### 2.8.3 Color Measurement

A Minolta Chroma Meter CR‐400 (Konica Minolta Co., Osaka, Japan) was used to measure the color of the camel milk powder. The colorimeter's sensor was positioned over the sample, and color parameters (such as lightness [L*], redness [a*] and yellowness [b*]) were measured at a temperature of 25°C. Average value of three random points on the surface of the sample were used.

#### 2.8.4 Bulk and Tapped Density

The bulk and tapped density were calculated using the procedure outlined by Affandi et al. (2017). To measure bulk density, 1 g of the camel milk powder was placed into a 10 mL measuring cylinder. Without tapping the cylinder, the amount of space occupied by the powder was measured. The tapped density was then determined by tapping the loaded cylinder 15 times from a height of 10 cm on a rubber plate, and recording the final volume. Preliminary tests showed that after 15 taps, the volume change was negligible, confirming that a constant volume was achieved. The bulk and tapped density were calculated using the following equations:

$$\text{Bulk density}\;\left(\rho_{B},g/{\mathrm{cm}}^{3}\right)=\text{Mass of the powder}/\text{Volume of the powder}$$

$$\text{Tapped density}\;\left(\rho_{T},g.{\mathrm{cm}}^{3}\right)=\text{Mass of the powder}/\text{Final tapped volume}$$

#### 2.8.5 Solubility Index

To evaluate solubility, 10 g of the sample was dissolved in 100 mL of distilled water by blending at maximum speed (200 rpm) of a magnetic stirrer for 5 min. A centrifuge (REMI R8C BL; ILE Company, Chennai) was used to centrifuge the mixture at 3000 rpm for 10 min. As described by Caparino et al. (2012), the supernatant was filtered, carefully transferred into a pre‐weighed aluminum dish and then dried in a hot air oven (FanAzma, Iran) at a temperature of 105°C ± 5°C for 5 h. The weight of the samples was measured every 2 h until a constant weight was reached. The solubility index is indicated by the weight of the solids in the dried supernatant as a percentage of the total dry solids in the original 1 g of the sample.

$$\text{Solubility index}\;\left(\%\right)=\text{Weight after centrifugation}/\text{Weight of initial sample}$$

#### 2.8.6 Sensory Evaluation

Sensory evaluation was conducted using a 5‐point hedonic scale, where 1 corresponded to “very undesirable” and 5 to “very desirable”. Eight trained panelists (4 men and 4 women) evaluated the camel milk powder samples for taste, aroma, color, and overall acceptability. Each panelist evaluated each sample once (*n* = 8 per sample).

For sample preparation, each camel milk powder sample was reconstituted based on a similar approach to that described by Kang et al. (2021), with minor modifications. Briefly, 1 g of powder was dissolved in 10 mL of distilled water at room temperature (25°C ± 2°C) to obtain a 10% (w/w) reconstituted milk, matching the natural total solids content of raw camel milk (Kang et al. 2021). Approximately 15 mL of each sample was served to panelists in labeled, odor‐free plastic cups. Three‐digit random codes were used to label the samples, and the order of presentation was randomized across panelists to avoid order bias.

After tasting each sample, panelists recorded their level of satisfaction on a provided evaluation form. To ensure accurate and consistent results, the tests were conducted under standardized conditions, including private tasting rooms with white lighting, and panelists were instructed to rinse their mouths with water between samples. Written informed consent was obtained from all participants prior to the tests. Participants were informed that their responses would be anonymous and that they could withdraw at any time. The study did not involve any harmful substances, and all samples were safe for human consumption.

### 2.9 Statistical Analysis

All data are presented as mean ± standard deviation (SD). No data transformation or normalization was applied. For powder properties, three independent powder batches were analyzed for each drying temperature (*n* = 3). One‐way analysis of variance (ANOVA) followed by Duncan's test (*α* = 0.05) was used to compare the means among the three drying temperatures (50°C, 60°C, and 70°C). Normality of residuals was confirmed by the Kolmogorov–Smirnov test (*p* > 0.05), and homogeneity of variances was confirmed by Levene's test (*p* > 0.05). All statistical analyses were performed using IBM SPSS statistical software version 22 (SPSS Inc., USA).

## 3 Results and Discussion

### 3.1 Fitting the Models

The density and stability of the formed foam are shown in Table 2. The selection of the most suitable model for each response variable (Foam density and Foam stability) was based on a combination of statistical parameters, including the sequential model sum of squares (Prob > *F*), the lack‐of‐fit test, the coefficients of determination (*R* ^2^ and adjusted *R* ^2^) and the predicted residual error sum of squares (PRESS).

#### 3.1.1 Foam Density

The quadratic model is recommended as the most appropriate. Although the linear model was significant (Prob > *F* = 0.0155), the quadratic model demonstrated a statistically superior fit. This is evidenced by a higher adjusted *R* ^2^ value (0.5446 for quadratic vs. 0.3682 for linear), indicating that the quadratic model explains a greater proportion of the variance in the data (Table 3). Furthermore, the lack‐of‐fit test for the quadratic model was not significant (*p* = 0.0637), confirming that the model is well‐suited to the data and that any error is due to random variation rather than an inadequate model (Table 3). The cubic model, while having a very high *R* ^2^, showed a non‐significant lack‐of‐fit but a markedly higher PRESS statistic (1.86 vs. 0.4729), suggesting a risk of overfitting and poorer predictive capability compared to the more parsimonious quadratic model (Table 3).

**TABLE 3:** ANOVA of response surface models for the density and stability of the formed foam.

Source	Foam density (g/cm^3^)			Foam stability (%)		
	Sum of squares	*F*‐value	Prob > *F*	Sum of squares	*F*‐value	Prob > *F*
(a) Model analysis						
Mean	10.38			4361.58		
Linear	0.130	0.0155	0.0155	1478.58	7.32	0.0026
2Fi	0.021	0.5627	0.5627	262.45	1.40	0.2881
Quadratic	0.060	0.0800	0.0800	343.31	2.43	0.1258
Cubic	0.046	0.0816	0.0816	126.74	0.55	0.7058
Residual	0.020			344.35		
Total	10.65			6917.02		
(b) Lack of fit						
Linear	0.135	5.46	0.1368	756.000	1.07	0.5041
2Fi	0.115	6.37	0.0783	493.55	0.96	0.5439
Quadratic	0.055	4.87	0.0637	150.24	0.47	0.7877
Cubic	0.008	3.71	0.1119	23.49	0.37	0.5716
Pure error	0.011			320.86		
(c) *R* ^2^ analysis	*R* ^2^	Adjusted *R* ^2^	PRESS	*R* ^2^	Adjusted *R* ^2^	PRESS
Linear	0.4680	0.3682	0.2616	0.5786	0.4996	1726.11
2Fi	0.5425	0.3313	0.4490	0.6813	0.5342	1709.99
Quadratic	0.7603	0.5446	0.4729	0.8156	0.6497	1672.06
Cubic	0.9288	0.7745	1.86	0.8652	0.5733	5640.16

#### 3.1.2 Foam Stability

Similarly, the quadratic model is selected for this response. The linear model was statistically significant (Prob > *F* = 0.0026) (Table 3). However, the quadratic model offers a superior fit with a significantly higher adjusted *R* ^2^ (0.6497 for quadratic vs. 0.4996 for linear). The lack‐of‐fit test for the quadratic model was not significant (*p* = 0.7877), validating its adequacy. The cubic model was excluded due to a lower adjusted *R* ^2^ (0.5733) and a drastically inflated PRESS value (5640.16), which indicates a poor ability to predict new observations (Table 3).

Based on the ANOVA results, the quadratic model was chosen for both foam density and foam stability. The models are statistically adequate, as indicated by the non‐significant lack‐of‐fit and satisfactory values of *R* ^2^ and adjusted *R* ^2^. The significance of the linear terms confirms that the process factors are key drivers of the responses, whereas the significance of the higher‐order terms allows for the precise mapping of the response surface to identify optimal processing conditions for the foam‐mat drying of camel milk powder.

### 3.2 Statistical Analysis of the Fitted Quadratic Models for Foam Properties

A quadratic model was developed to analyze the effects of three independent variables—concentration of foaming agent (A‐WPC), concentration of stabilizer (B‐GT), and whipping time (C‐MT)—on the responses of foam density and foam stability. The results of the Analysis of Variance (ANOVA) for the fitted models are presented in Tables 4 and 5. Due to the relatively low adjusted *R* ^2^ (0.5446) of the foam density model, indicating limited predictive power, the following discussion focuses primarily on the foam stability model (adjusted *R* ^2^ = 0.6497) and the drying kinetics.

**TABLE 4:** ANOVA of the fitted quadratic equations for the density (*Y*_1_) of the foam samples.

Source	Sum of squares	df	Mean squares	*F*‐value	Prob > *F*
Model	0.19	9	0.047	7.79	0.0013
A‐WPC	0.074	1	0.074	12.35	0.0031
B‐GT	2.860 × 10^−3^	1	2.860 × 10^−3^	0.43	0.5256
C‐MT	0.053	1	0.053	8.79	0.0096
AB	5.253 × 10^−3^	1	5.253 × 10^−3^	0.79	0.3937
AC	1.431 × 10^−3^	1	1.431 × 10^−3^	0.22	0.6518
BC	0.014	1	0.014	2.10	0.1783
A^2^	0.027	1	0.027	4.52	0.0501
B^2^	0.028	1	0.028	4.61	0.0484
C^2^	9.084 × 10^−5^	1	9.084 × 10^−5^	0.014	0.9090
Residual	0.066	10	6.613 × 10^−3^		
Lack of fit	0.055	5	0.011	4.87	0.0537
Pure error	0.011	5	2.255 × 10^−3^		
Total	0.28	19			
*R* ^2^	0.7603				
Adj. *R* ^2^	0.5446				

**TABLE 5:** ANOVA of the fitted quadratic equations for the stability (*Y*_2_) of the foam samples.

Source	Sum of squares	df	Mean squares	*F*‐value	Prob > *F*
Model	2084.35	9	231.59	4.92	0.0102
A‐WPC	710.50	1	710.50	15.08	0.0030
B‐GT	674.05	1	674.05	14.31	0.0036
C‐MT	94.03	1	94.03	2	0.1881
AB	189.15	1	189.15	4.02	0.0729
AC	1.90	1	1.90	0.040	0.8448
BC	71.40	1	71.40	1.52	0.2464
A^2^	116.67	1	116.67	2.48	0.1466
B^2^	91.76	1	91.76	1.95	0.1930
C^2^	112.37	1	112.37	2.39	0.1535
Residual	471.10	10	47.11		
Lack of fit	150.24	5	30.05	0.47	0.7877
Pure error	320.86	5	64.17		
Total	2554.44	19			
*R* ^2^	0.8156				
Adj. *R* ^2^	0.6497				

#### 3.2.1 Analysis of the Model for Foam Stability

As can be seen (Table 5), the developed quadratic model for foam stability was also statistically highly significant (*F*‐value = 4.92, *p*‐value = 0.0102). The model's *R* ^2^ value of 0.8156 and adjusted *R* ^2^ of 0.6497 demonstrate that it has good predictive capability and can be effectively used for optimization purposes.

For this response, the linear effects of both foaming agent concentration (A‐WPC) (*p*‐value = 0.0030) and stabilizer concentration (B‐GT) (*p*‐value = 0.0036) were highly significant (Table 5). This underscores the critical role of both ingredients in enhancing the stability of the foam. Conversely, the linear effect of whipping time (C‐MT), along with all interaction and quadratic terms, did not show a significant effect (*p*‐value > 0.05). This suggests that while the concentrations of WPC and GT are crucial, their interactions and non‐linear effects, as well as the whipping time, are less influential on stability within the tested ranges. The non‐significant lack of fit (*p*‐value = 0.7877) further validates the model's adequacy, indicating a good fit between the model predictions and the observed data. The effect of input variables on foam stability was explained by the below equation:

$$Y_{2}=10.7111+2.260A-81.754B$$

As a result, the ANOVA results confirm that the quadratic models are statistically significant and adequate for navigating the design space for both foam density and stability. The concentration of the foaming agent (WPC) was identified as the most critical factor, significantly affecting both responses. Whipping time was a key determinant for density, whereas the stabilizer concentration (GT) was paramount for achieving high foam stability. These findings provide a clear guide for optimizing the formulation and process parameters to produce foam with desired characteristics.

### 3.3 Analysis of Response Surfaces

To evaluate the impact of independent variables on dependent variables, surface response plots derived from quadratic polynomial models were created. These plots were generated by varying two independent variables within the experimental range while keeping another variable fixed at its central point.

#### 3.3.1 Foam Density

**FIGURE 1:**
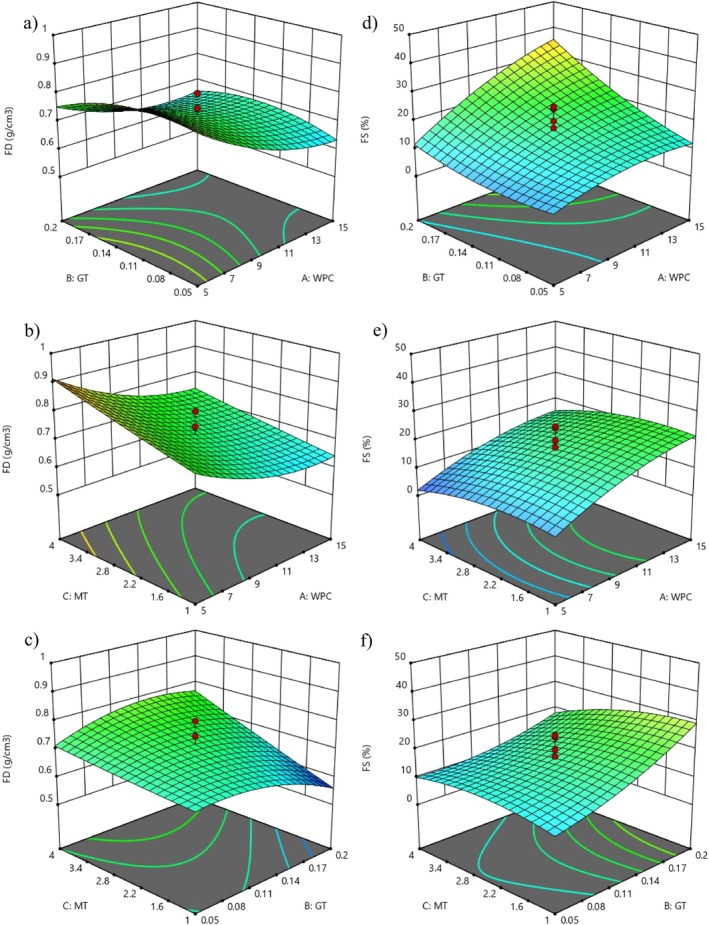
Response surface contour and 3D plots showing the effects of WPC concentration (%), GT concentration (%), and whipping time (min) on foam density (a–c) and foam stability (d–f). Each plot was derived from the central composite design (CCD) with 20 runs including six central point replicates. Statistical details of the models (ANOVA, *R* ^2^, adjusted *R* ^2^, lack‐of‐fit, *p*‐values) are provided in Tables 4 and 5. Raw data are presented in Table 2.

The combined effect of WPC and GT concentrations on foam density at a constant mixing time of 2.5 min is shown in Figure 1a. The contour plot demonstrates that foam density decreased with an increase in WPC concentration and a simultaneous decrease in GT concentration. The minimum foam density (approximately 0.5 g/cm^3^) was achieved at the highest level of WPC (~13%) and the lowest level of GT (0.05%). This indicates that protein is the primary driver for forming a low‐density, airy foam, whereas higher concentrations of gum tragacanth may inhibit optimal air incorporation, potentially by increasing the viscosity of the continuous phase.

The interaction between WPC concentration and mixing time on foam density at a fixed GT level of 0.125% is presented in Figure 1b. The results indicate that a high WPC concentration was the most critical factor for achieving low density. The minimal foam density (around 0.5 g/cm^3^) was obtained at the highest WPC level (~13%) within a broad optimal mixing time range of approximately 2.2–3.4 min. At shorter mixing times, insufficient air incorporation led to higher density, whereas at the longest time (4 min), a slight density increase suggests potential over‐whipping.

Figure 1c illustrates the effect of GT concentration and mixing time while the WPC content was held constant at 10%. The general trend clearly indicates that increasing the GT concentration led to a higher foam density, as the thickened continuous phase resisted efficient air entrapment. However, a minor deviation from this trend was observed at the combination of the highest GT level and the shortest mixing time, where the density was slightly lower. This apparent inconsistency may be attributed to experimental variability or a complex, non‐linear interaction at the extreme levels of these parameters. Despite this observation, the overall model conclusively shows that gum tragacanth increases foam density and that extended mixing times cannot compensate for its thickening effect.

These results are consistent with previous findings. Dabestani and Yeganehzad (2019) reported that the addition of Persian gum and xanthan gum to egg white increased its viscosity and consequently raised foam density (Dabestani and Yeganehzad 2019). Similarly, Izadi et al. (2020) demonstrated that the use of WPC as a foaming agent in the drying of white cheese reduced foam density, confirming WPC's effectiveness in generating light, aerated foams (Izadi et al. 2020). In another study, Salahi and Mohebbi (2021) also found that WPC reduced foam density while stabilizing soy milk foam, whereas hydrocolloid gums (xanthan, guar, and locust bean) increased foam density due to higher viscosity (Salahi and Mohebbi 2021). In a related study, Kamali Sarvestani et al. (2021) observed similar opposing effects of WPC and xanthan gum on foam density during the foam‐mat drying of celery juice powder. Collectively, these studies confirm the inverse relationship between foaming agent concentration and foam density, and the direct relationship between gum concentration and foam viscosity (Kamali Sarvestani et al. 2021).

#### 3.3.2 Foam Stability

The effect of WPC and GT concentrations on foam stability at a constant mixing time is shown in Figure 1d. The region of maximum foam stability (approximately 35%–40%) is located where both WPC and GT are at their highest levels (~13% and 0.15%, respectively). This indicates a synergistic effect between the two components; the WPC provides the primary stabilizing film at the interface, whereas the high concentration of GT likely enhances stability by significantly increasing the bulk viscosity, thereby retarding drainage and coalescence more effectively than at lower concentrations as previously reported for Arabic gum and xanthan (Abd Karim and Wai 1999). Comparable results were observed by Febrianto et al. (2012), who demonstrated that increasing gum Arabic concentration improved the stability of milk foams during foam‐mat drying (Febrianto et al. 2012). Likewise, Salahi and Mohebbi (2021) found that xanthan, guar, and locust bean gums markedly increased soy milk foam stability. The present findings corroborate these studies, emphasizing the dual contribution of WPC and GT in achieving high foam stability through protein–polysaccharide interactions that enhance interfacial film strength (Salahi and Mohebbi 2021).

The interaction between WPC concentration (A) and mixing time (C) on foam stability at a fixed GT (B) level is presented in Figure 1e. The highest stability (approximately 40%) was achieved at the highest WPC concentration (13%) with an intermediate mixing time of around 3.4 min. This suggests that while high protein content is essential, an optimal mixing duration is required to achieve maximum stability. The decrease in stability at the longest mixing time (4 min) reinforces the hypothesis of over‐whipping, which can disrupt the foam structure.

Figure 1f illustrates the effect of GT concentration (B) and mixing time (C) while the WPC content (A) was held constant. The results demonstrate that foam stability increases with both GT concentration and mixing time, reaching a maximum (≥ 40 min) at the highest levels of both factors (GT = 0.14%, mixing time = 4 min). This strong positive correlation confirms that in the presence of a constant, moderate amount of protein, gum tragacanth plays a dominant role in stabilizing the foam, and sufficient mixing time is critical to fully integrate its thickening and stabilizing properties throughout the system.

### 3.4 Drying Kinetics

The drying behavior of camel milk foam at 50°C, 60°C, and 70°C was best described by the Page model, which exhibited the highest coefficient of determination (*R* ^2^ > 0.99) and the lowest root mean square error (RMSE < 0.02) (Table 6). These results confirm the suitability of the Page model for describing thin‐layer drying kinetics. Comparable outcomes have been reported in several studies. For example, Kashaninejad et al. (2022) evaluated eight empirical models for drying camel milk cream foam and identified the Page and Midilli models as the most accurate (Kashaninejad et al. 2022). Inyang et al. (2018) reviewed 33 drying models and found the Page model to be the most widely applicable for food materials (Inyang et al. 2018). Similarly, Ghaboos et al. (2016) reported that the Page model best described the drying kinetics of pumpkin juice (Ghaboos et al. 2016). Increasing the drying temperature accelerated moisture removal, which aligns with findings by Azizpour et al. (2016), who observed faster drying rates and lower equilibrium moisture contents at elevated temperatures.

**TABLE 6:** Fitting parameters of thin‐layer drying kinetic models for foam‐mat dried camel milk at different temperatures.

Models	Drying temperature (°C)		
	50	60	70
*Page*			
*K*	0.0613	0.0462	0.0676
*N*	0.7722	0.9379	1.0275
*R* ^2^	**0.9977**	**0.9983**	**0.9984**
RMES	**0.0116**	**0.0118**	**0.0131**
*Louis*			
*K*	0.0249	0.0371	0.0730
*N*	—	—	—
*R* ^2^	0.9718	0.9970	0.9982
RMES	0.0398	0.0154	0.0132
*Henderson and pubis*			
*K*	0.8898	0.9761	1.0093
*N*	0.219	0.0362	0.0737
*R* ^2^	0.9861	0.9977	0.9984
RMES	0.0283	0.0138	0.0134

*Note:* Bold values indicate the best‐fitting model (highest R^2^ and lowest RMSE) selected for describing the drying kinetics of camel milk foam.

Overall, the results demonstrate that the Page model provides an excellent fit for the drying kinetics of camel milk foam, reflecting the exponential moisture reduction characteristic of foam‐mat drying. The observed increase in drying rate with temperature can be attributed to the enhanced vapor pressure gradient and reduced product viscosity at elevated temperatures, resulting in improved mass transfer efficiency.

### 3.5 Evaluating the Foaming Properties

#### 3.5.1 Water Activity (*a*_w_)

The a_w_ of the foam‐mat dried camel milk powder was significantly influenced by the drying temperature. As presented in Table 7, the *a*_w_ values decreased with increasing drying temperature, from 0.205 at 50°C to 0.136 at 70°C. This reduction is critical for powder stability, as lower *a*_w_ inhibits microbial growth and extends shelf life, aligning with findings in other foam‐mat dried products such as cantaloupe pulp (Salahi et al. 2017) and aquafaba (Aslan and Ertaş 2021). A recent study on foam‐mat dried tropical red fruit blend powder also confirmed that higher drying temperatures lead to significantly lower *a*_w_, enhancing product stability (Paiva et al. 2023).

**TABLE 7:** Physical properties of camel milk powder dried by foam‐mat drying.

Temperature (°C)	*a*_w_	Density (g/cm^3^)		Hygroscopicity (%)	Solubility (%)	Color		
		Bulk	Tapped			L*	a*	b*
50	0.205 ± 0.01^a^	0.49 ± 0.02^a^	0.71 ± 0.01^a^	9.02 ± 0.12^c^	0.83 ± 0.24^b^	87.78 ± 0.19^b^	0.84 ± 0.09^b^	19.19 ± 0.59^b^
60	0.147 ± 0.03^b^	0.50 ± 0.00^a^	0.66 ± 0.01^b^	9.28 ± 0.16^b^	0.87 ± 0.19^a^	89.3 ± 0.00^a^	0.64 ± 0.03^c^	18.08 ± 0.01^c^
70	0.136 ± 0.02^c^	0.45 ± 0.01^b^	0.61 ± 0.03^c^	9.94 ± 0.09^a^	0.88 ± 0.13^a^	89.39 ± 0.11^a^	1.18 ± 0.02^a^	19.86 ± 0.02^a^

*Note:* Values are expressed as mean ± standard deviation (*n* = 3). Three independent powder batches were analyzed for each drying temperature. Different superscript letters within each column indicate significant differences (*p* < 0.05).

#### 3.5.2 Bulk and Tapped Densities

Both bulk and tapped densities of the camel milk powder decreased as the drying temperature increased. Bulk density ranged from 0.49 g/cm^3^ at 50°C to 0.45 g/cm^3^ at 70°C, whereas tapped density varied between 0.71 g/cm^3^ (50°C) and 0.61 g/cm^3^ (70°C). The inverse relationship between temperature and density can be attributed to rapid moisture removal, which promotes the formation of a more porous structure. This is consistent with the principle that water, being denser than dry solids, contributes to higher density at higher moisture levels (Nejatdarabi and Mohebbi 2021). Similar trends were reported by Kamali Sarvestani et al. (2021) for celery juice powder, where increased drying temperature reduced both bulk and tapped densities due to decreased residual moisture (Kamali Sarvestani et al. 2021). Recent work on tomato powder also demonstrated that low‐density, high‐porosity structures formed at higher temperatures improve reconstitution properties (Hossain et al. 2024).

#### 3.5.3 Hygroscopicity

Hygroscopicity refers to the tendency of a food powder to absorb moisture from the surrounding environment when the ambient relative humidity exceeds its equilibrium moisture content. This property is critical as it directly influences the physical, chemical, and microbiological stability of the powder, making its understanding essential for determining appropriate drying, packaging, and storage conditions (Franco et al. 2016).

As shown in Table 7, hygroscopicity values ranged from 9.02% (50°C) to 9.94 g % (70°C), indicating a slight increase with temperature. This may be due to structural changes and increased surface area at higher temperatures (see Section 3.5.2), facilitating moisture absorption. The quality of the powder can be considered acceptable, as its hygroscopicity remains close to the benchmark of < 10% (or 10 g/100 g) recommended for high‐quality dairy powders (Indrianti et al. 2023). The slight exceedance can be attributed to the inherent composition of camel milk, particularly its lactose content and soluble proteins, which contain hydrophilic groups that bind to moisture. Similar trends of increasing hygroscopicity with temperature have been observed in other foam‐mat dried products like cantaloupe, kiwiberry and aquafaba pulps (Aslan and Ertaş 2021; Bogusz et al. 2024; Salahi et al. 2017), likely due to structural changes that increase surface area and amorphous regions, thereby enhancing water absorption.

#### 3.5.4 Color

Color parameters (L*, a*, b*) of the camel milk powder were affected by drying temperature. Lightness (*L*) increased from 87.78 (50°C) to 89.39 (70°C), indicating that higher temperatures produced a lighter powder. This is likely due to shorter drying times, which limit non‐enzymatic browning reactions. The a* values (red‐green axis) were relatively low across samples, with a slight increase at 70°C, whereas b* values (yellow‐blue axis) remained stable, suggesting minimal browning. These results align with studies on foam‐mat dried banana pulp (Kamali et al. 2022) and egg white foam (Dabestani and Yeganehzad 2019), where higher temperatures preserved lightness by reducing Maillard reaction time. Recent research on milk protein foams also emphasizes that rapid drying at 70°C helps maintain light color and prevents thermal degradation of spinach powder (Emami et al. 2024).

#### 3.5.5 Solubility

Solubility is a crucial quality attribute for instant food powders. According to Table 7, solubility increased with drying temperature, from 0.83 at 50°C to 0.88 at 70°C. This improvement is attributed to the formation of a more porous structure at higher temperatures. Elevated temperatures shorten the drying time, which helps preserve the integrity of the foam's air bubbles by minimizing their collapse. This retained porosity enhances water penetration and dissolution during reconstitution. Similar mechanisms have been reported in other foam‐mat dried products; for instance, in cheese powder (Izadi et al. 2020) and tomato powder (Hossain et al. 2024), where increased drying temperature led to better‐preserved porosity and consequently higher solubility.

#### 3.5.6 Sensory Evaluation

The sensory evaluation results for the foam‐mat dried camel milk powders are summarized in Table 8. Statistical analysis revealed no significant differences (*p* > 0.05) among the samples dried at 50°C, 60°C, and 70°C for any of the assessed attributes—taste, aroma, color, and overall acceptance. This indicates that varying the drying temperature within this range did not detrimentally affect the sensory profile perceived by the trained panelists. Therefore, all samples were sensorially acceptable.

**TABLE 8:** Sensory evaluation of the foam‐mat dried camel milk.

Temperature (°C)	Organoleptic characteristics			
	Taste	Aroma	Color	Overall acceptance
50	4.12 ± 0.83^a^	4.25 ± 0.89^a^	4.12 ± 0.64^a^	4.12 ± 0.35^a^
60	4.5 ± 0.53^a^	4.37 ± 0.52^a^	3.87 ± 0.99^a^	4.00 ± 1.1^a^
70	4.6 ± 0.74^a^	4.87 ± 0.35^a^	3.62 ± 0.74^a^	4.12 ± 1.1^a^

*Note:* Values are expressed as mean ± standard deviation (*n* = 8). Eight trained panelists evaluated each sample once. Different superscript letters within each column indicate significant differences (*p* < 0.05).

Although no statistically significant differences were found, the physicochemical analysis showed that the powder dried at 70°C exhibited the lowest water activity (0.136), highest solubility (0.88), and greatest lightness (L* = 89.39). These improved physicochemical properties, combined with the sensory acceptability of all samples, support the selection of 70°C as a technically favorable drying temperature for producing camel milk powder.

## 4 Conclusion

This study demonstrated the feasibility of producing camel milk powder with favorable physicochemical and functional properties using the foam‐mat drying technique. Through Response Surface Methodology, the foam formulation was optimized, with whey protein concentrate concentration identified as the critical factor controlling both foam density and stability. The drying kinetics of the optimized camel milk foam were accurately described by the Page model across all tested temperatures (50°C, 60°C, and 70°C).

The drying temperature significantly influenced the final powder properties. Powder produced at 70°C exhibited superior quality attributes, including the lowest water activity (0.136), which is critical for microbial stability and extended shelf life, and the highest solubility (0.88), indicative of excellent reconstitution potential. This was accompanied by a lower bulk density and a lighter color, suggesting a more porous structure and minimal thermal degradation. Although hygroscopicity slightly increased with temperature, the values remained close to the acceptable benchmark for dairy powders.

Notably, sensory evaluation indicated that all powders were acceptable, with no statistically significant differences attributed to drying temperature. However, a consistent trend showed the highest mean scores for taste and overall acceptance were given to the powder dried at 70°C, aligning with its superior instrumental profile.

In conclusion, foam‐mat drying at 70°C is recommended as an effective processing method for camel milk. It yields a powder with optimal physicochemical stability, functional properties, and sensory acceptability. This technique presents a scalable, energy‐efficient alternative to traditional drying methods, facilitating the broader commercialization and utilization of camel milk as a valuable functional food ingredient. To further enhance the value of the product, future research should focus on the retention of heat‐sensitive bioactive compounds (e.g., immunoglobulins, vitamin C) during the foam‐mat drying process and the application of the optimized powder in specific functional food formulations.

## Author Contributions

**Mohammd Nejatian:** conceptualization, supervision, project administration, writing – review and editing, funding acquisition. **Morteza Fathi:** supervision, writing – review and editing, methodology. **Maryam Taghdir:** methodology, supervision, writing – review and editing. **Najmeh Youseftabar Miri:** formal analysis, methodology, writing – original draft. **Mohamad Ali Podine:** writing – original draft, formal analysis, investigation.

## Consent

Sensory evaluation was performed by eight trained panelists. Written informed consent was obtained from all participants prior to the tests. Participants were informed that their responses would be anonymous and that they could withdraw at any time. The study did not involve any harmful substances, and all samples were safe for human consumption.

## Conflicts of Interest

The authors declare no conflicts of interest.

## Data Availability

The data that support the findings of this study are available from the corresponding author upon reasonable request.

## References

[fsn372084-bib-0001] Abd Karim, A. , and C. C. Wai . 1999. “Foam‐Mat Drying of Starfruit ( *Averrhoa carambola* L.) Puree. Stability and Air Drying Characteristics.” Food Chemistry 64, no. 3: 337–343.

[fsn372084-bib-0002] Affandi, N. , W. Zzaman , T. A. Yang , and A. M. Easa . 2017. “Production of *Nigella sativa* Beverage Powder Under Foam Mat Drying Using Egg Albumen as a Foaming Agent.” Beverages 3, no. 1: 9.

[fsn372084-bib-0003] Agrawal, R. , D. Kochar , M. Sahani , F. Tuteja , and S. Ghorui . 2004. “Hypoglycemic Activity of Camel Milk in Streptozotocin Induced Diabetic Rats.” International Journal of Diabetes in Developing Countries 24: 47–49.

[fsn372084-bib-0004] Alhassani, W. E. 2024. “Camel Milk: Nutritional Composition, Therapeutic Properties, and Benefits for Human Health.” Open Veterinary Journal 14, no. 12: 3164–3180. 10.5455/OVJ.2024.v14.i12.2. 39927355 PMC11799641

[fsn372084-bib-0005] Aslan, M. , and N. Ertaş . 2021. “Foam Drying of Aquafaba: Optimization With Mixture Design.” Journal of Food Processing and Preservation 45, no. 3: e15185.

[fsn372084-bib-0006] Azizpour, M. , M. Mohebbi , and M. H. H. Khodaparast . 2016. “Effects of Foam‐Mat Drying Temperature on Physico‐Chemical and Microstructural Properties of Shrimp Powder.” Innovative Food Science & Emerging Technologies 34: 122–126.

[fsn372084-bib-0007] Bakry, I. A. , L. Yang , M. A. Farag , et al. 2021. “A Comprehensive Review of the Composition, Nutritional Value, and Functional Properties of Camel Milk Fat.” Food 10, no. 9: 2158. 10.3390/foods10092158 PMC8472115 34574268

[fsn372084-bib-0008] Bogusz, R. , M. Nowacka , K. Rybak , D. Witrowa‐Rajchert , and E. Gondek . 2024. “Foam‐Mat Freeze Drying of Kiwiberry ( *Actinidia arguta* ) Pulp: Drying Kinetics, Main Properties and Microstructure.” Applied Sciences 14, no. 13: 5629.

[fsn372084-bib-0009] Caparino, O. A. , J. Tang , C. I. Nindo , S. S. Sablani , J. R. Powers , and J. K. Fellman . 2012. “Effect of Drying Methods on the Physical Properties and Microstructures of Mango (Philippine ‘Carabao’ Var.) Powder.” Journal of Food Engineering 111, no. 1: 135–148. 10.1016/j.jfoodeng.2012.01.010.

[fsn372084-bib-0010] Chikha, M. , and B. Faye . 2025. “Camel Milk: White Gold and Its Contribution to the Sustainable Development Goals—A Review.” Outlook on Agriculture 54, no. 1: 42–54.

[fsn372084-bib-0011] Dabestani, M. , and S. Yeganehzad . 2019. “Effect of Persian Gum and Xanthan Gum on Foaming Properties and Stability of Pasteurized Fresh Egg White Foam.” Food Hydrocolloids 87: 550–560.

[fsn372084-bib-0012] Doymaz, I. 2006. “Drying Kinetics of Black Grapes Treated With Different Solutions.” Journal of Food Engineering 76, no. 2: 212–217.

[fsn372084-bib-0013] Emami, M. S. , M. Mohebbi , and M. Khalilian‐Movahhed . 2024. “An Investigation Into Properties of Foam‐Mat‐Dried Spinach Powder and Physical Properties of Spinach Cube.” Journal of Food Processing and Preservation 2024, no. 1: 9114105.

[fsn372084-bib-0014] Febrianto, A. , S. Kumalaningsih , and A. W. Aswari . 2012. “Process Engineering of Drying Milk Powder With Foam Mat Drying Method. A Study on the Effect of the Concentration and Types of Filler.” Journal of Basic and Applied Research International 2, no. 4: 3588–3592.

[fsn372084-bib-0015] Franco, T. S. , C. A. Perussello , L. N. Ellendersen , and M. L. Masson . 2016. “Effects of Foam Mat Drying on Physicochemical and Microstructural Properties of Yacon Juice Powder.” LWT‐Food Science and Technology 66: 503–513.

[fsn372084-bib-0016] Gebrehiwot, H. H. , and F. Banat . 2025. “Powdered Camel Milk Production and Changes Occurring Along the Processing Steps and Storage Conditions: A Comprehensive Review.” Food Chemistry 493: 146050. 40896999 10.1016/j.foodchem.2025.146050

[fsn372084-bib-0017] Ghaboos, S. H. H. , S. M. S. Ardabili , M. Kashaninejad , G. Asadi , and M. Aalami . 2016. “Combined Infrared‐Vacuum Drying of Pumpkin Slices.” Journal of Food Science and Technology 53, no. 5: 2380–2388. 27407204 10.1007/s13197-016-2212-1 PMC4921089

[fsn372084-bib-0018] Hatami, M. , M. Nejatian , and M. A. Mohammadifar . 2012. “Effect of Co‐Solute and Gelation Temperature on Milk Protein and Gum Tragacanth Interaction in Acidified Gels.” International Journal of Biological Macromolecules 50, no. 4: 1109–1115. 22405780 10.1016/j.ijbiomac.2012.02.026

[fsn372084-bib-0019] Hatami, M. , M. Nejatian , M. A. Mohammadifar , and H. Pourmand . 2014. “Milk Protein–Gum Tragacanth Mixed Gels: Effect of Heat‐Treatment Sequence.” Carbohydrate Polymers 101: 1068–1073. 24299875 10.1016/j.carbpol.2013.10.004

[fsn372084-bib-0020] He, J. , K. Guo , Q. Chen , Y. Wang , and Jirimutu . 2022. “Camel Milk Modulates the Gut Microbiota and Has Anti‐Inflammatory Effects in a Mouse Model of Colitis.” Journal of Dairy Science 105, no. 5: 3782–3793. 10.3168/jds.2021-21345. 35248376

[fsn372084-bib-0021] Henderson, S. , and S. Pabis . 1961. “Grain Drying Theory. 1. Temperature Effect on Drying Coefficient.” Journal of Agricultural Research Engineering 6: 169–174.

[fsn372084-bib-0022] Ho, T. M. , S. Chan , A. J. E. Yago , R. Shravya , B. R. Bhandari , and N. Bansal . 2019. “Changes in Physicochemical Properties of Spray‐Dried Camel Milk Powder Over Accelerated Storage.” Food Chemistry 295: 224–233. 10.1016/j.foodchem.2019.05.122. 31174753

[fsn372084-bib-0023] Hossain, M. A. , T. Ahmed , J. Ferdaus , and W. Zzaman . 2024. “Optimization of the Foam‐Mat Drying Process to Develop High‐Quality Tomato Powder: A Response Surface Methodology Approach.” Heliyon 10, no. 21: e39811. 39559223 10.1016/j.heliyon.2024.e39811 PMC11570490

[fsn372084-bib-0024] Ibrahim, A. , and S. Khalifa . 2015. “Effect of Freeze‐Drying on Camel's Milk Nutritional Properties.” International Food Research Journal 22, no. 4: 1438–1445.

[fsn372084-bib-0025] Indrianti, N. , D. P. Putri , L. E. Yulianti , et al. 2023. “Optimization of Foam Properties and Evaluation of the Drying Temperature Effects on the Foam‐Mat Drying of Pasta Sauce.” Agricultural Technology 43, no. 3: 238–250.

[fsn372084-bib-0026] Inyang, U. E. , I. O. Oboh , and B. R. Etuk . 2018. “Kinetic Models for Drying Techniques—Food Materials.” Advances in Chemical Engineering and Science 8, no. 2: 27–48.

[fsn372084-bib-0027] Izadi, Z. , M. Mohebbi , F. Shahidi , M. Varidi , and M. R. Salahi . 2020. “Cheese Powder Production and Characterization: A Foam‐Mat Drying Approach.” Food and Bioproducts Processing 123: 225–237.

[fsn372084-bib-0028] Kamali, R. , S. Dadashi , J. Dehghannya , and H. Ghaffari . 2022. “Numerical Simulation and Experimental Investigation of Foam‐Mat Drying for Producing Banana Powder as Influenced by Foam Thickness.” Applied Food Research 2, no. 1: 100075.

[fsn372084-bib-0029] Kamali Sarvestani, M. , M. Mohebbi , and M. Taghizadeh . 2021. “Optimization of Foam Production in Foam Mat Drying of Celery Juice and Evaluation of Its Powder Properties.” Iranian Food Science and Technology Research Journal 17, no. 2: 217–231. 10.22067/ifstrj.2020.39265.

[fsn372084-bib-0030] Kang, M. W. , D. Chen , R. Ruan , and Z. M. Vickers . 2021. “The Effect of Intense Pulsed Light on the Sensory Properties of Nonfat Dry Milk.” Journal of Food Science 86, no. 9: 4119–4133. 34383322 10.1111/1750-3841.15865

[fsn372084-bib-0031] Kashaninejad, M. , S. M. A. Razavi , and M. R. Salahi . 2022. “Drying Kinetics of Camel Milk Cream Foam Using Foam Mat Drying and Study Its Effect on the Structure and Color of the Product.” Iranian Food Science and Technology Research Journal 18, no. 1: 65–79.

[fsn372084-bib-0032] Kaskous, S. 2016. “Importance of Camel Milk for Human Health.” Emirates Journal of Food and Agriculture 28, no. 3: 158–163.

[fsn372084-bib-0033] Kinsella, J. E. 1981. “Functional Properties of Proteins: Possible Relationships Between Structure and Function in Foams.” Food Chemistry 7, no. 4: 273–288. 10.1016/0308-8146(81)90033-9.

[fsn372084-bib-0034] Nejatdarabi, S. , and M. Mohebbi . 2021. “Effect of Foam‐Mat Drying Condition on Physical Properties and Rehydration Behavior of Mushroom Powder.” Iranian Food Science & Technology Research Journal 17, no. 3: 1.

[fsn372084-bib-0035] Nejatdarabi, S. , K. Parastouei , and M. Fathi . 2023. “Development of Ajwain ( *Trachyspermum ammi* ) Seed Essence Powder Using Foam‐Mat Drying Technique: A Comparison on the Effect of Guar Gum, Basil Seed Gum, and the Combination of Them.” Journal of Food Measurement and Characterization 17, no. 1: 75–86. 10.1007/s11694-022-01597-7.

[fsn372084-bib-0036] Paiva, Y. F. , R. M. F. D. Figueirêdo , A. J. D. M. Queiroz , et al. 2023. “Tropical Red Fruit Blend Foam Mat Drying: Effect of Combination of Additives and Drying Temperatures.” Food 12, no. 13: 2508. 10.3390/foods12132508 PMC10340195 37444246

[fsn372084-bib-0037] Rakhmatulina, A. , F. Dikhanbayeva , D. Tlevlessova , et al. 2024. “Advancements in Camel Milk Drying Technology: A Comprehensive Review of Methods, Chemical Composition, and Nutritional Preservation.” Dairy 5, no. 3: 360–371.

[fsn372084-bib-0038] Salahi, M. R. , and M. Mohebbi . 2021. “Development of Soy Milk in the Form of Wet Foam in the Presences of Whey Protein Concentrate and Polysaccharides at Different Whipping Temperatures: Study of Physical, Rheological and Microstructural Properties.” LWT 137: 110444.

[fsn372084-bib-0039] Salahi, M. R. , M. Mohebbi , and M. Taghizadeh . 2017. “Development of Cantaloupe ( *Cucumis melo* ) Pulp Powder Using Foam‐Mat Drying Method: Effects of Drying Conditions on Microstructural of Mat and Physicochemical Properties of Powder.” Drying Technology 35, no. 15: 1897–1908.

[fsn372084-bib-0040] Sangamithra, A. , S. Venkatachalam , S. G. John , and K. Kuppuswamy . 2015. “Foam Mat Drying of Food Materials: A Review.” Journal of Food Processing and Preservation 39, no. 6: 3165–3174. 10.1111/jfpp.12421.

[fsn372084-bib-0041] Sarsavadia, P. , R. Sawhney , D. Pangavhane , and S. Singh . 1999. “Drying Behaviour of Brined Onion Slices.” Journal of Food Engineering 40, no. 3: 219–226.

[fsn372084-bib-0042] Swelum, A. A. , M. T. El‐Saadony , M. Abdo , et al. 2021. “Nutritional, Antimicrobial and Medicinal Properties of Camel's Milk: A Review.” Saudi Journal of Biological Sciences 28, no. 5: 3126–3136. 10.1016/j.sjbs.2021.02.057. 34025186 PMC8117040

[fsn372084-bib-0043] Thuy, N. M. , N. N. N. Minh , N. H. Kha , et al. 2025. “Application of Foam‐Mat Drying to Produce Field Crab Powder: Foaming Process Optimization, Drying Kinetics, and Final Product Characterization.” Journal of Agriculture and Food Research 22: 102047.

